# Tegument Ultrastructure and Morphology of the Capsule Surrounding the Tetrathyridia of the Genus *Mesocestoides* Vaillant, 1863 in the Liver of the Root Vole

**DOI:** 10.1134/S0012496623700461

**Published:** 2023-10-13

**Authors:** N. A. Pospekhova, K. V. Kusenko

**Affiliations:** Institute of Biological Problems of the North, Far Eastern Branch, Russian Academy of Sciences, Magadan, Russia

**Keywords:** *Mesocestoides*, tetrathyridium, ultrastructure, extracellular vesicles, shed microtriches, capsule, host–parasite interaction

## Abstract

The ultrastructure of the tegument of encapsulated tetrathyridia of the genus *Mesocestoides* Vaillant, 1863 (Cestoda, Cyclophyllidea, Mesocestoididae) from the liver of root voles *Microtus oeconomus* (Pallas, 1776) and the structure of the three-layered capsule surrounding them were studied for the first time. Several types of extracellular structures were noted on the surface of the tetrathyridia tegument: vesicles, fine granular material, and vacuoles. In addition, the phenomenon of shedding microtriches, which have expanded parts, was found. Host cells in contact with extracellular material show signs of destruction. A characteristic feature of the capsules surrounding the tetrathyridia is the reticular structure of the fibrous layer containing both native and degenerating inflammatory cells.

Cestodes of the genus *Mesocestoides* use predatory mammals (foxes, arctic foxes, wolves, and dogs) as definitive hosts; intermediate hosts are more diverse and vary from amphibians to primates [1–6]. In the Northeast of Russia, rodents and insectivores are intermediate hosts of *Mesocestoides* [7, 8]. Tetrathyridia (the metacestode stage of this parasite) have a characteristic appearance; however, the absence of the rostellum hampers their identification to the species level [3, 8–10]. The current classification system for metacestodes defines the tetrathyridium as an “alacunar form with inverted scolex” [11]. The majority of cyclophyllid metacestodes have protective envelopes differing in number, structure, and origin [12], whereas the members of the genus *Mesocestoides* do not have such membranes, the scolex and neck are invaginated inside the tetrathyridium, and the hindbody tegument contacts the host tissues [13].

Features of the tegument morphology and parasite–host interactions of cyclophyllid metacestodes (including *Mesocestoides* tetrathyridia) are the subject of numerous studies [13–24]. However, most of these studies were performed on laboratory cultures of cestodes and host animals, whereas in this work we studied the morphology of the contact area between tetrathyridia of the genus *Mesocestoides* and liver tissues of a naturally infected host, the root vole *Microtus oeconomus* (Pallas, 1776) from the tundra ecosystem of Chukotka. Since the life cycle of representatives of the genus *Mesocestoides* has not yet been established [1, 25], it is not possible to indicate the source of infection in voles.

## MATERIALS AND METHODS

Root voles were caught in the vicinity of the Chaunskii Station of the Institute of Biological Problems of the North of the Far Eastern Branch of the Russian Academy of Sciences (Northwestern Chukotka, Russia). When whitish spots were found in the liver tissue of one of the voles, capsules were excised from the liver, and some of them were opened. Tetrathyridia were found in the cavity of the opened capsules; the average size of the capsules was 2.5–3.0 mm. Unopened capsules were excised from the host liver together with surrounding tissue and fixed in 2% glutaraldehyde solution in 0.1 M phosphate buffer (pH 7.2) at ~4°C for subsequent electron microscopic examination. After fixation in glutaraldehyde, the material was postfixed in a 2% OsO_4_ solution in 0.2 M phosphate buffer (pH 7.2) for 12 h, dehydrated, and embedded in EPON–araldite mixture. During dehydration, the samples were stained with a saturated solution of uranyl acetate in 70% ethanol overnight. Semithin sections, which were obtained using LKB Bromma 2088 and LKB Nova microtomes (Sweden), were stained with methylene blue according to Morgenstern [26] and viewed under an Olympus CX41 microscope (Olympus Corporation, Japan) equipped with an Olympus E-420 digital camera. Ultrathin sections (90 nm) cut on an LKB ultratome (Sweden) were viewed using JEM-1011 and JEM-1400Plus (JEOL, Japan) transmission electron microscopes at 80 kV.

## RESULTS AND DISCUSSION

### Light Microscopy

**Fig. 1.**
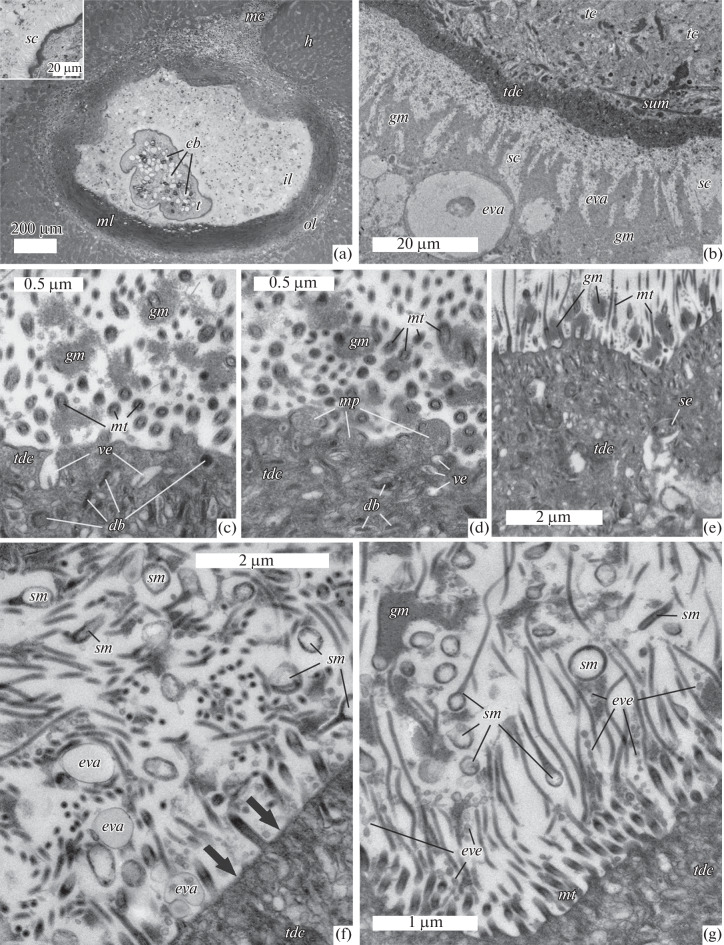
Encapsulated *Mesocestoides* tetrathyridium and ultrastructure of its tegument: (a) semithin section of a capsule with tetrathyridium in the liver tissue; inset, “solar crown;” (b) tetrathyridium tegument with microtriches and fine-granular material; (c) distal cytoplasm of the tegument with dense bodies and vesicles; (d) matrix protrusion of the distal cytoplasm; (e) tetrathyridium tegument with sensory ending; (f) microtrichial border with extracellular vacuoles, fine granular material, and shed microtriches (arrows indicate the areas devoid of microtriches); (g) microtrichial border with extracellular vesicles and shed microtriches. Designations: *bl—*basal lamina, *cb—*calcareous bodies, *db—*dense bodies, *eva—*extracellular vacuoles, *eve—*extracellular vesicles, *ger—*granular endoplasmic reticulum, *gm—*granular material, *h—*hepatocyte, *hc—*host cell, *il—*inner layer of capsule, *mc—*migration channel of tetrathyridium, *ml—*middle layer of capsule, *mp—*matrix protrusion of distal cytoplasm, *mt—*microtriches, *ol—*outer layer of capsule, *sc—*“solar crown”, *se—*sensory ending, *sm—*shed microtrix, *sum—*subtegumental muscle, *t—*tetrathyridium, *tc—*tegumental cyton; *tdc—*tegument distal cytoplasm.

A semithin section of a capsule with a tetrathyridium in the host liver tissue is shown in Fig. 1a. The migratory passage of the tetrathyridium to the localization site is filled with host cells, among which granular leukocytes can be distinguished. The capsule consists of three layers. The first, outer layer adjoins host hepatocytes and consists of cells of moderate electron density, among which leukocytes predominate. This layer is thickest near the migratory passage and becomes thinner as the distance from it increases. The second, middle, fibrous layer is infiltrated with leukocytes, its most distant part from the migratory passage has the highest density of fibers and the smallest number of leukocytes. The fibrous layer is 120 to 280 µm thick. The space between the fibrous layer and the tetrathyridium surface is filled with host cells of different morphology, which can be considered as the third, inner layer of the capsule. The contact area between the tetrathyridium and the inner layer of the capsule has different morphology in different areas, which is clearly seen when viewing a series of tangential sections of a capsule with a tetrathyridium. On the sections that cut off a small sector of the metacestode (Fig. 1a), its surface is separated from the host cells by a light zone that looks like a “solar crown” (Fig. 1a, inset). The thickness of this zone varies from 15 to 50 µm. In the areas of direct contact between the parasite microtriches and the host cells, the light zone disappears and mechanical deformation of the microtrichial border is observed. The dense distal cytoplasm of the tegument and the large (up to 15 μm in diameter) calcareous bodies in the tetrathyridium subtegument are the most noticeable details of the parasite structure at the light-optical level.

### Electron Microscopy

**Tegument of the hindbody of** ***Mesocestoides*** **tetrathyridium***.* The teguments of the studied tetrathyridia consist of the distal cytoplasm of the tegument covered with microtriches, basal plate, subtegumental musculature, and underlying tegument cytons (Fig. 1b). The thickness of the distal cytoplasm ranges from 3 to 10 µm, the basal plate is approximately 300 nm thick. Cytons of the tegument with spherical nuclei and light cytoplasm are located in 1–2 layers under the subtegumental muscles. The latter is represented by superficial circular and deeper longitudinal bundles. The distal cytoplasm of the metacestode tegument (Figs. 1c–1e) contains numerous dense rod-shaped and disc-shaped bodies approximately 250 nm in diameter. Rod-shaped and disc-shaped bodies with exfoliated outer membrane are regularly found. In such bodies, a narrow light zone between the contents and the outer membrane is observed, and the contents seem heterogeneous in density.

Numerous oval vesicles up to 400 nm in length usually contain granular material of moderate electron density or look “empty.” A characteristic feature is the location of the “empty” vesicles near the outer membrane; in rare cases they are open to the surface of the tegument (Fig. 1c).

The outer membrane of the distal cytoplasm of the tetrathyridium tegument in some areas has small (up to 500 nm in diameter) protrusions containing the distal cytoplasm matrix (Fig. 1d). Sometimes such protrusions are fairly large (up to 4 µm in diameter).

In the distal cytoplasm thickness, non-ciliated sensory endings were noted (Fig. 1e). Tegumental microtriches are of the same type, with a short (approximately 500 nm) basal part and a dense whip-shaped apical part, which reaches 15 µm in length. At the outer surface of the distal cytoplasm of the hindbody tegument, between the microtriches, various types of extracellular membrane structures and granular material were noted.

The fine granular material near the tegument surface is represented by separate accumulations 300–400 nm in diameter, which often adjoin microtriches (Figs. 1c–1e). Closer to the surface, small portions of granular material fuse and divide the microtrichial border into separate bundles, which creates the effect of a “solar crown.” Outside the microtrichial border, the granular material forms large clusters contacting the host cells or their fragments (Fig. 1b).

Membrane structures are represented by small (30–60 nm) extracellular vesicles and extracellular vacuoles 150–500 nm in size (Figs. 1f, 1g). Vesicles are often arranged in chains oriented perpendicular to the tegument surface (Fig. 1g). The diameter of extracellular vacuoles increases with distance from the surface of the distal cytoplasm (Fig. 1f). Outside the microtrichial border, vacuoles larger than 20 µm in diameter were noted (Fig. 1b).

In addition to the three described types of material, which is recorded near the distal cytoplasm surface, we found a large number of shed microtriches, which occurred regularly but without a pronounced dependence on the contact area morphology. In the microtrichial border thickness, above the bases of microtriches, spherical vacuole-like structures with a dense shell 200–250 nm in diameter (sometimes up to 500 nm in diameter) were scattered. These structures are shed microtriches with expanded (swollen) areas, which, depending on the plane of the cut, may look spherical, oval, or may continue to form a dense flagelliform structure similar to the apical part of the tegument microtriches of the posterior part of the tetrathyridium (Figs. 1f, 1g). In addition, we observed fragments of microtrichial bases located at a distance of up to 5 µm from the distal cytoplasm surface. In the zones of mass “secretion” of microtriches, small surface areas free of microtriches were noted; however, we did not find any signs of damage to the outer membrane (Fig. 1f, arrows).

### Contact Zone

**Fig. 2.**
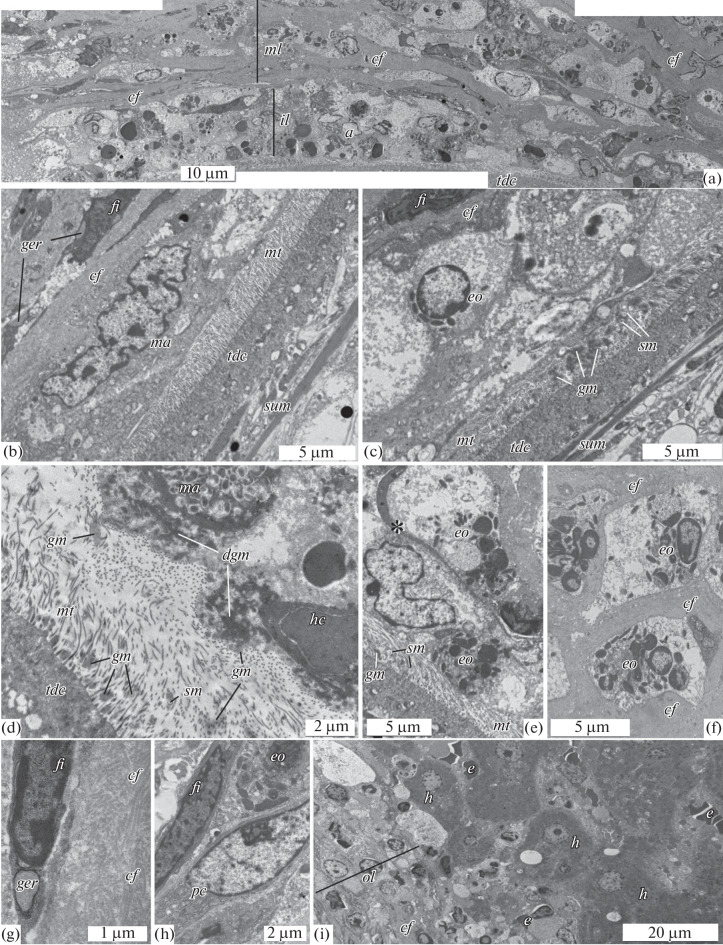
Ultrastructure of the capsule around *Mesocestoides* tetrathyridium: (a) capsule fragment (inner and part of the middle layer); (b) macrophage at the surface of the tetrathyridium tegument; (c) degrading eosinophil in the contact area; (d) two types of granular material at the surface of host cells; (e) fibroblast process (*) in the inner layer of the capsule; (f) fragment of the fibrous layer with degrading cells; (g) fibroblast and collagen fibers; (h) cells in the fibrous layer; (i) outer layer of the capsule and liver tissue. Designations; *a—*cells with signs of apoptosis*, cf—*collagen fibers, *dgm*—dense granular material, *e—*erythrocyte, *eo*—eosinophil, *fi*—fibroblast, *ger—*granular endoplasmic reticulum, *gm*—fine-granular material, *h*—hepatocyte, *hc*—host cell, *il*—inner layer of capsule, *ma—*macrophage; *ml*—middle layer of capsule, *mt*—microtriches, *ol*—outer layer of capsule, *pc*—plasma cell; *sum*—subtegumental muscle; *tc*—tegumental cyton; *tdc*—distal cytoplasm of tegument.

The contact area of the tetrathyridium with the host includes the inner layer of the capsule, the parasite tegument together with its outgrowths (microtriches) and extracellular material, as well as the space between them, which is usually filled with cell detritus. The width of this zone varies depending on the tetrathyridium position in the capsule cavity. When the surface of the distal cytoplasm of the tetrathyridium is located at a large distance from the inner layer of the capsule, microtriches, together with fine granular material, form a “solar crown,” the area mentioned above, which passes into clusters of the same material with large extracellular vacuoles (Fig. 1b). Host cells contacting the granular material show signs of damage: their cytoplasm is vacuolated, the cytoplasmic membrane integrity is disturbed, and granules and lipid droplets are released into the surrounding space. Cells with signs of apoptosis (condensed chromatin in the nuclei and fragmentation of the nucleus and cells) were noted (Fig. 2a). The majority of cells in the contact area, judging by the characteristic structure of the granules, can be attributed to eosinophils, although a significant number of macrophages were also noted.

In the zone of direct contact between the host cells  and the tetrathyridium surface, mechanical deformation of the microtrichial border was observed (Figs. 2b, 2c, 2e). In this case, the distance between the distal cytoplasm surface and the cells of the inner layer of the capsule decreases to 1.5–2.0 µm. Clusters of fine granular material and single shed microtriches (Figs. 2c–2e) are constantly present among the microtriches. Host cells in such zones may not have visible damage (Fig. 2b); the microtrichial border adjoining this cell does not contain fine granular material. Partially or completely degenerated cells usually adjoin the microtriches, among which clusters of fine granular material can be seen (Figs. 2c–2e). Fine granular material sometimes forms local clusters near the host cell surface (Fig. 2d); it is located outward of the granular material of higher electron density, which adjoins the cytoplasmic membrane of host cells.

At the boundary of the inner and middle (fibrous) layers of the capsule, we regularly found fibroblasts with long narrow outgrowths that extended into the contact area, closer to the damaged cells (Fig. 2e, asterisk).

### Middle Layer of the Capsule

The middle (fibrous) layer of the capsule is a reticular structure formed by multidirectional bundles of collagen fibers, in which granulocytes (mainly eosinophils), macrophages, and plasma cells are located (Figs. 2a, 2f–2h). As a rule, these cells show varying degrees of degradation; however, there are also cells without visible damage. Fibroblasts accompanying collagen fibers show signs of synthetic activity, in particular, dilated canals of the granular endoplasmic reticulum (Figs. 2b, 2c, 2g). We noted differences in the packing density of collagen fiber bundles in the fibrous layer of the capsule: the density increased with distance from the tetrathyridium migratory path along with a decrease in the number of cells (or their fragments) in the layer.

### Outer Layer of the Capsule

The outer layer of the capsule is formed primarily by inflammatory cells; however, fibroblasts and small-section collagen fibers are regularly found between the layers of these cells (Fig. 2i). Hepatocytes in contact with the outer layer of the capsule contain vacuoles with flocculent material in their cytoplasm. Large cavities containing vesicles, vacuoles, flocculent material, and membrane fragments adjoin hepatocytes. Numerous capillaries with erythrocytes are located among hepatocytes near the outer layer of the capsule but are absent in the composition of the capsule wall.

Capsule formation in the intermediate host is a characteristic feature of the development of tapeworms at the metacestode stage [14, 16, 18, 20, 27–30]. Moreover, according to some authors [18, 27, 31], capsules always form around the larvocysts of higher cestodes (order Cyclophyllidea). Capsules, regardless of the type of tissue in which they are located (muscular or connective), have a two-layer structure: the inner layer is formed by fibrous structures, whereas the outer layer is enriched in cellular elements.

The capsules surrounding the tetrathyridia in the root vole liver look three-layered, and even the middle, fibrous layer is rich in cellular elements, both intact and at different stages of degradation. Of interest is the reticular structure of the fibrous layer, which is clearly seen during electron microscopic examination and is poorly distinguishable in the light-optical preparations. This reticular structure is probably formed as a result of isolation of degrading (and possibly intact) immunocompetent cells by the fibroblast activity products. This assumption is confirmed by the constant presence of fibroblast processes near the parasite–host contact area. The presence of fibroblasts and collagen fibers in the outer layer of the capsule suggests that the fibrous layer of the capsule grows both from the inside and from the outside. Berezan-tsev et al. [18] described a “developed hemicirculatory network” consisting of three layers in the walls of the capsule around the *Hydatigera taeniaeformis* (Batsch, 1786) metacestode. In our case, we found no capillaries in the capsule wall, although it cannot be ruled out that the capsules around the tetrathyridia in root voles undergo vascularization at later stages of development of parasite–host relations.

Since our material was taken from the liver of naturally infected voles, we can judge about the duration of the invasion only by the indirect signs and literature data. For example, Berezantsev et al. [18] showed that capsules around taeniid cysticerci become 100–150 µm thick 50 days after infection. Capsules around *Mesocestoides* tetrathyridia in the liver of albino mice are assessed as “well-defined” only 1 year after experimental infection of the intermediate host, in contrast to those not yet formed 44 days after infection [14]. However, noticeable encapsulation with collagen deposition was observed as early as on day 35 after the infection of albino mice with *Mesocestoides* tetrathyridia, and on day 60 their liver showed extensive regeneration around the encapsulated tetrathyridia [17]. Apparently, at least 1 month has passed since the infection of root voles.

The structure of the capsules around helminths in intermediate and paratenic hosts has different characteristics. For example, the acanthocephalan larvae in the paratenic hosts form capsules of three types: fibroblastic, leukocytic, and intermediate [32]. According to this classification, the capsules around the tetrathyridia in root voles can be classified as intermediate, because they contain both fibroblasts, along with the products of their activity (collagen fibers), and leukocytes, which are more often represented by granulocytes. Cells with signs of apoptosis in the zone of contact with the host were also recorded by other authors [33], which is interpreted as one of the ways by which the parasite suppresses the cellular immune response of the host.

Granulocytes (mostly eosinophils) were constantly mentioned in descriptions of the host response to the presence of helminth larvae (in particular, taeniid metacestodes). Different authors not only noted the presence of eosinophils near the surface of the parasite tegument but also described the processes of degranulation, as well as destruction of these cells with the release of granules into the contact area [16, 20, 28, 34, 35]. The same authors note that the host cells that are in contact with the parasite surface undergo degradation and destruction, whereas the parasite itself in most cases remains undamaged. The causes for this stability probably lie in the protective properties of the tegumentary structures of metacestodes (in this case, the tetrathyridium hindbody tegument). The morphology and ultrastructure of the tegument of *Mesocestoides corti*/*vogae* tetrathyridium [36], a laboratory culture of budding metacestodes [15, 37, 38] have been studied most comprehensively. These authors divide the tetrathyridium tegument into three regions depending on the predominant types of microtriches, with only filamentous microtriches being observed at the posterior end of the body [37]. We have designated such microtriches as flagelliform, taking into account the density and length of the apical end. In accordance with the international nomenclature of microtriches, this type is classified with filitrichs (namely, capilliform filitriches) [39]. Several types of extracellular material were found between microtriches, part of which (or all types, as we assume) are secretory products of the tegument.

The secretory activity of the tegument of cestodes is noted at all stages of the life cycle, both in nature and in experiment [22, 40–46]; the nature of tegumental secretion may vary significantly. For example, in *Hymenolepis diminuta* (Rudolphi, 1819), two different subtypes of small vesicles were noted, including those in the form of chains [46]. The authors attribute the appearance of vesicles to the release of the contents of dense bodies outside the distal cytoplasm, and their arrangement in the form of chains is explained by their movement among microtriches.

In developing ascocerci from dragonfly larvae, microtriches of the outer tegument of the exocyst are buried in the surface layer, which consists of fine granular material, vesicles, and fibrils oriented parallel to the distal cytoplasm surface [45]. The authors believe that the components of the surface layer are synthesized by cytons of the exocyst tegument, whereas the layer itself is analogous to the glycocalyx and performs protective functions.

Fine-granular material in the form of small clusters up to 400 nm in diameter, which is often present between the microtriches of encapsulated tetrathyridium, structurally resembles the granular matrix of the distal cytoplasm. This material is most likely released to the surface by lacing off protrusions of the distal cytoplasm, followed by destruction of their boundary membrane. In addition, it was assumed that the contents of dense disc-shaped and rod-shaped bodies undergoes morphological changes, after which it is either released to the tegument surface by the merocrine pathway to form a glycocalyx or serves to replenish the distal cytoplasm matrix, which is consumed during the parasite life activity [37, 47, 48].

It should be noted that material morphologically similar to fine granular material was interpreted in some papers as a product of degranulation of granulocytes adjacent to the tegument [16] or as a precipitate formed on the surface of microtriches as a result of exposure to the host immune serum [42, 49]. The appearance of shed microtriches, which, as in our case, looked like vacuoles with dense walls [42] but were located in a layered manner over the microtrichial border, was also attributed to the effect of serum. The layered arrangement of the material secreted by the tegument of cestodes was noted in several species [42, 45, 50]. Most clearly it is represented in cystic echinococci in the form of a laminated layer surrounding the cyst [51]. Apparently, the initial template for layered protective structures on the surface of parasitic worms at different developmental stages is their glycocalyx, which in most cases is at least bilayer [52].

Despite the methodological complexity of study, the excretory–secretory processes in helminths draw particular attention, given the possibility of using the results of such studies for practical purposes [46, 50, 53–56]. The authors of some of the above-cited works pay special attention to the extracellular vesicles released from the surface of parasites, and it cannot be ruled out that that the chains of vesicles noted by us carry substances with immunomodulatory properties.

The morphological features of the contact area between tetrathyridia and host tissues (presence or absence of fine granular material, vesicles, and vacuoles, distance to host cells, and degree of degradation of these cells) were not constant, possibly due to the movement of tetrathyridium in the capsule and the creation of temporary local sites of close interaction of parasite surfaces and the host. The asymmetry of the reaction around the parasites was noted earlier. Intact and damaged inflammatory cells, cell debris, eosinophil granules, and parts of microvilli were noted on one side of the *T. taeniaeformis* cysticercus in the rat liver, whereas on the opposite pole the surface of the parasite was in contact with normal hepatocytes [20].

The analysis of the morphology of the host–parasite interaction between the tetrathyridia of the genus *Mesocestoides* and root voles allowed us to note several features: the three-layer capsule consisting of the middle fibrous and two (inner and outer) leukocyte layers; the reticular structure of the fibrous layer, which, apparently, grows both from the outside and from the inside; and, finally, the mass shedding of expanded microtriches, which does not lead to disruption of the outer membrane of the tegument. The last phenomenon was previously described in the experiment when the tegument was exposed to immune serum [42], whereas in our case it was observed in a naturally infected host.
