# Erratum to: **Tegument Ultrastructure and Morphology of the Capsule Surrounding the Tetrathyridia of the Genus** ***Mesocestoides*** **Vaillant, 1863 in the Liver of the Root Vole**

**DOI:** 10.1134/S001249662305006X

**Published:** 2024-01-25

**Authors:** N. A. Pospekhova, K. V. Kusenko

**Affiliations:** Institute of Biological Problems of the North, Far Eastern Branch, Russian Academy of Sciences, Magadan, Russia

The article “Tegument Ultrastructure and Morphology of the Capsule Surrounding the Tetrathyridia of the Genus *Mesocestoides* Vaillant, 1863 in the Liver of the Root Vole,” written by N.A. Pospekhova and K.V. Kusenko, was originally published electronically in Springer-Link on October 13, 2023 without Open Access. After publication in volume 511, pages 213–221, the authors decided to make the article an Open Access publication. Therefore, the copyright of the article has been changed to © The Author(s) 2023 and the article is forthwith distributed under the terms of a Creative Commons Attribution 4.0 International License (http://creativecommons.org/licenses/by/4.0/, CC BY), which permits use, duplication, adaptation, distribution and reproduction of a work in any medium or format, as long as you cite the original author(s) and publication source, provide a link to the Creative Commons license, and indicate if changes were made.

